# Comparison of EBRT and I-125 seed brachytherapy concerning outcome in intermediate-risk prostate cancer

**DOI:** 10.1007/s00066-021-01815-z

**Published:** 2021-08-05

**Authors:** Matthias Moll, Andreas Renner, Christian Kirisits, Christopher Paschen, Alexandru Zaharie, Gregor Goldner

**Affiliations:** 1 Department of Radiation Oncology, Medical University of Vienna, Vienna, Austria; 2 Division of Nephrology and Dialysis, Department of Medicine III, Medical University of Vienna, Vienna, Austria

**Keywords:** Biochemical control, bNED, Toxicity, Side effects, Favourable intermediate risk

## Abstract

**Purpose:**

This study’s objective was the comparison of external beam radiotherapy (EBRT) and I‑125 seed brachytherapy regarding clinical outcome and development of side effects.

**Patients and methods:**

In all, 462 localized intermediate-risk prostate cancer patients treated between 2000 and 2019 at our department using either I‑125 seed brachytherapy or EBRT with a dose of 74 or 78 Gy were included: 297 patients were treated with EBRT and 165 with seeds. Biochemical no evidence of disease (bNED) rates according to Phoenix definition as well as late gastrointestinal and urogenital side effects (EORTC/RTOG) were assessed.

**Results:**

Patients were followed up yearly with a median follow-up of 54 (3–192) months. Observed bNED rates for 74 Gy, 78 Gy and seeds were 87, 92, and 88% after 5 years and 71, 85, and 76% after 9 years, respectively. No significant differences were found comparing seeds with 74 Gy (*p* = 0.81) and 78 Gy (*p* = 0.19), as well as between 74 and 78 Gy (*p* = 0.32). Concerning gastrointestinal side effects, EBRT showed significantly higher rates of RTOG grade ≥ 2 toxicity compared to seeds, but at no point of the follow-up more than 10% of all patients. However, genitourinary side effects were significantly more prevalent in patients treated with seeds, with 33% RTOG grade ≥ 2 toxicity 12 months after treatment. Nevertheless, both types of side effects decreased over time.

**Conclusion:**

Favorable intermediate-risk prostate cancer patients can be treated either by external beam radiotherapy (74/78 Gy) or permanent interstitial seed brachytherapy.

## Introduction

Localized primary prostate cancer can be treated via external beam radiotherapy (EBRT) or permanent interstitial seed brachytherapy (BT). Both of these treatment modalities achieve excellent tumor control rates [1–3]. The recommendations and guidelines consider these modalities as equivalent especially for patients with low-risk prostate cancer [1, 2]. For intermediate-risk patients this strict recommendation to perform seeds BT is missing [1]. Nevertheless, numerous studies evaluating the tumor control rates after seeds BT included besides low-risk prostate cancer also intermediate-risk patients and reported excellent tumor control rates [4–11]. However, up to now no randomized trial successfully evaluating the effectiveness of seeds BT compared to EBRT has been published so far. The German PREFERE trial [12], coming closest, tried to compare the outcomes of active surveillance, EBRT, BT and prostatectomy in low- and favorable intermediate-risk prostate cancer, but recruited only 345 out of the targeted 7600 patients and was therefore closed, thus, leaving the question unanswered by a prospective study. Our objective with this study is to compare both methods with data acquired from clinical routine patients treated at our department over a period of 20 years. For the evaluation we use data from intermediate-risk prostate cancer patients. We report the results concerning biochemical no evidence of disease (bNED) as well as late gastrointestinal und genitourinary side effects, as increased bNED rates shift the focus on side effects.

## Patients and methods

The study protocol was approved by the ethical review board of our medical university according to local law regulations (EK no. 1991/2019).

All patients included were treated at our Department of Radiation Oncology and had to meet the following inclusion criteria:

- Intermediate-risk primary prostate cancer as defined by the NCCN classification [13]: Prostate-specific antigen (PSA) between 10 and 20 ng/ml, or Gleason score 7, or a TNM stage of T2b or T2c,
- Favorable intermediate risk: only one or two risk factors regarding T‑stage, PSA or Gleason score,
- Localized cancer with a clinical stage of cNx/0 and cMx/0,
- Primary treatment locally limited to the prostate, and
- EBRT patients treated from 2000 to 2015 and I‑125 seed BT patients treated from 2004–2019.

Intermediate-risk prostate cancer showing the characteristics above were in general candidates for either EBRT or seeds BT. Both treatment options were discussed with the patient. The final treatment decision was left to the patient.

I‑125 seeds were transperineally implanted as a monotherapy. Dose prescription was 145 Gy for the prostate surrounding isodose according to the TG 137 protocol [14]. The source strength was on average 0.57 µGy × m^2^/h per seed. All seed applications were performed by one single radiation oncologist. The intervention was performed using spinal anesthesia and patients stayed in hospital for 3 days.

For EBRT, from 2000 to 2009, the prescribed dose was 74 Gy with 2 Gy per fraction and patients were treated by three-dimensional (3D) conformal four field box radiotherapy. All patients received a rectal balloon for internal immobilization [15] and the safety margin around the clinical target volume was 10 mm in all directions for the first 66 Gy followed by 8 Gy with reduced dorsal margin of 5 mm.

For EBRT from 2010 to 2015 dose was escalated to 78 Gy with 2 Gy per fraction using either 3D conformal radiotherapy or volume modulated arc therapy (VMAT) from 2013 onwards. Again, all patients received a rectal balloon and in addition prior to radiotherapy gold marker fiducials were implanted. Due to the long time frame of our study, safety margins varied over time. The safety margin around the clinical target volume was 5 mm in all directions with gold marker fiducials, 7 mm in all directions without for 78 Gy, and 10 mm in the 74 Gy group for the first 66 Gy and 5 mm for the last 8 Gy.

Hormonal therapy was recommended for patients receiving EBRT for a duration of 6 months. However, the prescription of hormonal therapy was in the hands of the referring urologists and a certain proportion of patients refused hormonal therapy.

bNED failure was defined as PSA nadir +2 ng/ml using the Phoenix criteria [16]. Follow-up after treatment was scheduled after 3 months, 12 months, and every year from that point on. For each follow-up, gastrointestinal and genitourinary side effects were prospectively assessed using RTOG grading [17], and PSA levels were documented. All measures of time were calculated from the last day of radiotherapy.

Statistical analysis was performed using GraphPad Prism 9 (GraphPad Software, San Diego, CA, USA) and SPSS 26 (IBM, Armonk, NY, USA). All statistical tests were two-sided and a *p*-level < 0.05 was considered statistically significant. The Kaplan–Meier method was used to estimate bNED rates. The resulting curves were compared using the log-rank test. The univariate Cox regression model included the following variables: T‑stage, PSA, Gleason score, androgen deprivation therapy (ADT) and treatment modality. For all analyses, Gleason score (0 = “2–6” vs. 1 = “7”), T‑stage (0 = “T1a–c and 2a” vs. 1 = “T2b/c”), hormonal therapy (0 = “no” vs. 1 = “yes”), risk groups (0 = “one risk factor”, 1 = “2 risk factors”) and dose (0 = “74 Gy” vs 1 = “78 Gy” and 2 = “seeds”) were treated as categorical variables with the lowest category serving as reference category, while PSA was treated as an ordinal variable. Side effects were analyzed using the Mann–Whitney U test. Internal testing regarding the difference between 74 and 78 Gy over the course of 10 years was performed. Within this time span, the only significant differences we found were 36 and 72 months in concerning gastrointestinal side effects. Therefore, we merged both EBRT groups. Side effects are arranged in a group with RTOG grade 0 and 1 and another one with RTOG grade 2 and higher. Thereby, we aim to provide a better overview of the level of occurrence of clinically relevant side effects.

## Results

Our retrospective analysis included 462 primary intermediate-risk prostate cancer patients with a median follow-up of 54 (3–192) months: 297 patients received EBRT, 185 with a total dose of 74 Gy and 112 with a total dose of 78 Gy. Furthermore, 165 patients were treated with seeds. Further relevant patient characteristics are summarized in Table 1.

**Table 1:** Patient characteristics

	74 Gy	%	78 Gy	%	I‑125 seeds	%
*n* =	185		112		165	
T‑stage						
*1a/b*	10	5	5	4	0	0
*1c*	93	50	57	51	113	68
*2a*	29	16	13	12	29	18
*2b/c*	53	29	37	33	23	14
iPSA in ng/ml						
*Min*	1.98	–	1.5	–	1.7	–
*Max*	19.90	–	19.9	–	19.0	–
*Median*	10.2	–	7.4	–	7.2	–
Gleason score						
*<* *6 or 6*	94	51	47	42	58	35
*7a*	36	19	40	36	70	42
*7b*	18	10	24	21	26	16
*7 not otherwise specified*	37	20	1	1	11	7
ADT						
*Yes*	119	64	40	36	31	19
*Median duration in months*	10	–	11	–	7	4
Age during treatment in years						
*Min*	54	–	47	–	49	–
*Max*	85	–	83	–	86	–
*Median*	73	–	74	–	69	–
Follow-up						
*Min*	3	–	3	–	3	–
*Max*	192	–	108	–	166	–
*Median in months*	60	–	48	–	50	–
Technique						
*Seeds*	0	0	0	0	165	100
*3D conformal*	183	99	66	59	0	0
*VMAT or IMRT*	2	1	46	41	0	0
Risk group factor						
*1*	131	71	85	76	155	94
*2*	54	29	27	24	10	6

*ADT* androgen deprivation therapy, *IMRT* intensity-modulated radiation therapy, *VMAT* volumetric modulated arc therapy

**Fig. 1:**
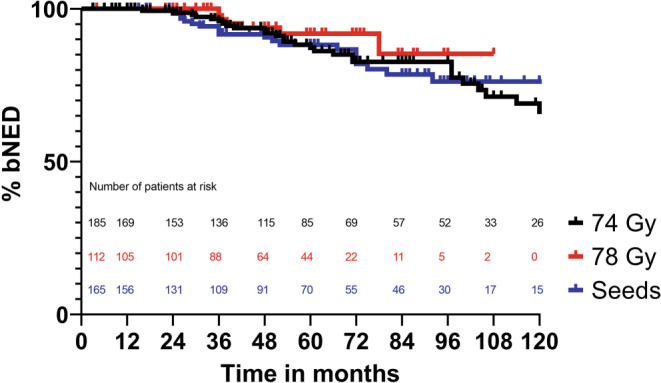
bNED after either EBRT (with 74 or 78 Gy) or seeds. *P*-value = 0.32 comparing 74 and 78 Gy, *p* = 0.81 comparing 74 Gy and seeds, *p* = 0.19 comparing 78 Gy and seeds

Concerning the treatment modalities, most notable differences are the lower rate of Gleason score 6 patients and the lower percentage of ADT in BT patients compared to EBRT. The median follow-up was longer for patients treated with 74 Gy (60 months), as the application of 78 Gy. Furthermore, 47% of all patients had a follow-up of at least 60 months. As of 2019, 60% of all patients were lost to follow-up.bNED rates for 74 Gy, 78 Gy, and seeds are displayed in Fig. 1. For 74 Gy, bNED rates after 5 and 9 years were 87% and 71%. The latest reported bNED failure occurred after 126 months. In the 78 Gy group, corresponding bNED rates were 92% after 5 years and 85% after 9 years, respectively. For seeds, the bNED rates were 88% after 5 years and 76% after 9 years. Comparing the Kaplan–Meier curves, 74 Gy vs 78 Gy and seeds vs 74 or 78 Gy showed no significance (*p* = 0.32, 0.81, and 0.19, respectively).

Table 2 displays the results of the univariate analysis. Significant influences regarding the bNED outcome were only detectable in patients receiving ADT. This result was also confirmed in a log-rank test, comparing all patients who received ADT with those who did not (*p* = 0.04).

**Table 2:** Univariate analysis of bNED

	*p*	HR	Lower 95% CI	Upper 95% CI
T2b/c vs T1a–c and 2a	0.10	1.60	0.92	2.78
Gleason score 7 vs 6 and < 6	0.89	0.96	0.57	1.63
Initial PSA	0.43	1.03	0.96	1.10
2 risk factors vs 1	0.11	1.60	0.90	2.86
ADT applied	0.04	0.57	0.33	0.98
Age	0.18	0.97	0.93	1.01
78 Gy vs 74 Gy	0.22	0.59	0.25	1.37
Seeds vs 74 Gy	0.81	0.93	0.53	1.65
Seeds vs 78 Gy	0.20	1.76	0.74	4,22

*ADT* androgen deprivation therapy

For further analysis, we compared patients who received ADT vs patients who did not within each group. For 74 and 78 Gy, no significant differences were found (*p* = 0.91 and 0.23). For BT, we discovered a significant advantage for ADT (*p* = 0.005), without a single bNED failure for patients receiving ADT over a median of 84 months, but within a small collective of 31 patients. As we discovered ADT as the only significant factor in our univariate analysis, no further multivariate analyses were performed.

Maximum late side effects at any point during treatment and follow-up are displayed in Table 3. Overall, all our subgroups tolerated the treatment well, with no more than 34% of any grade 2 toxicity in any group. Furthermore, patients receiving EBRT report in 61% of the cases no gastrointestinal and in 50% no genitourinary side effects. For seeds, 65% of all patients report no gastrointestinal side effects, but only 10% report no genitourinary side effects. There were 3 patients suffering from grade 4 toxicity reported. One patient treated with 78 Gy developed a fistula and needed a transient stoma that was surgically removed after 6 months. Two patients treated with seeds developed RTOG grade 4 genitourinary toxicity in the form of urinary retention that required operative urological intervention. Comparing the data regarding side effects from grade 0 to 4, there was a significant difference between 74 Gy and 78 Gy groups concerning maximum genitourinary side effects (*p* = 0.04). No difference was found for maximum gastrointestinal side effects in EBRT (*p* = 0.14). Comparing EBRT with seeds, we observed a borderline significant difference (*p* = 0.06) regarding maximum late gastrointestinal and a significant difference regarding genitourinary toxicity (*p* = 0.06 and *p* < 0.001, respectively).

**Table 3:** Maximum of late gastrointestinal and genitourinary side effects

Maximum of	Gastrointestinal side effects				Genitourinary side effects			
RTOG	74 Gy	78 Gy	EBRT	Seeds	74 Gy	78 Gy	EBRT	Seeds
Grade 0	64%	55%	61%	65%	54%	43%	50%	10%
Grade 1	15%	21%	17%	26%	23%	23%	23%	18%
Grade 2	19%	22%	20%	8%	17%	28%	21%	67%
Grade 3	2%	2%	2%	0%	6%	6%	6%	4%
Grade 4	0%	1%	0%	0%	0%	0%	0%	1%
*n*	185	111	296	165	185	111	296	165

**Fig. 2:**
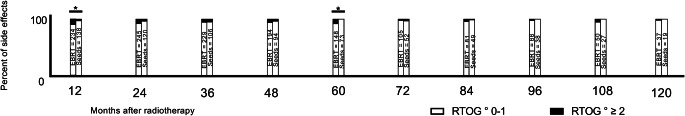
Development of gastrointestinal side effects after treatment with EBRT or seeds over a follow-up period of 120 months (*p < 0.05, **p < 0.01, ***p < 0.001)

**Fig. 3:**
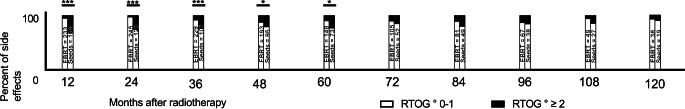
Development of genitourinary side effects after treatment with EBRT or seeds over a follow-up period of 120 months (*p < 0.05, **p < 0.01, ***p < 0.001)

The course of side effects over a follow-up period of 120 months is displayed in Figs. 2 and 3. Internal testing regarding the difference between 74 and 78 Gy over the course of 10 years was performed. Side effects are arranged in a group with RTOG grade 0 and 1 and another one with RTOG grade 2 and higher. Thereby, we aim to provide a better overview of the level of occurrence of clinically relevant side effects.

For the first and fifth year of follow-up, we observed a significantly higher level of gastrointestinal RTOG grade ≥ 2 toxicity in patients treated with EBRT. Nevertheless, the highest rate of RTOG grade ≥ 2 toxicity was only observed in 10% of patients treated with EBRT after 12 months of follow-up. From this point on, higher levels of gastrointestinal toxicity declined over time and were almost gone after 120 months of follow-up.

Genitourinary RTOG grade ≥ 2 toxicity was significantly higher in patients treated with seeds up to a maximum of 33% after 12 months of follow-up. While, just like gastrointestinal side effects, also declining over time, 11% of all seeds patients reported RTOG grade ≥ 2 toxicity after 120 months of follow-up. For EBRT, RTOG grade ≥ 2 toxicity alternated between 3% and 13% of all patients without a decline over time. Differences regarding the number of patients at risk between bNED rate and toxicity are due to lack of documented PSA and toxicity.

## Discussion

Intermediate-risk prostate cancer can be equally effectively treated either via radical prostatectomy or EBRT [13, 18–20]. The role of BT in these patients is seen critically, especially in the German S3 guideline, although several studies show good biochemical control rates in intermediate-risk prostate cancer patients [4–11, 21]. A strict recommendation for permanent interstitial low dose rate (LDR)-BT is limited to low-risk patients. Up to now, no randomized trial comparing LDR-BT versus EBRT versus radical prostatectomy was successfully completed—regardless of patients’ prostate cancer risk. The ratio to restrict the recommendation for LDR-BT to the low-risk group is their excellent tumor control rates. Nevertheless, various studies reporting about LDR-BT also included beside low-risk patients, intermediate-risk patients with reliable proportion and respectable tumor control rates. Due to the lack of randomized trials, retrospective analysis including a sufficient proportion of patients treated within a center of excellence by EBRT with a dose corresponding to the dose recommended within the guidelines (at least 74 Gy) or LDR-BT are important.

Concerning our bNED rates in seeds patients, the results of 88% bNED after 5 years and 76% after 10 years are within range of rates reported in the literature. A comparison of EBRT and seed BT published by Goldner et al. in 2012 [4] showed a bNED rate after 5 years of 75% for EBRT with 74 Gy and 81% for seeds. Now, we are able to display a bNED rate of 87% after 5 years for 74 Gy and 88% for seeds, respectively. With this data, we are able to recreate the lack of significant differences between 74 Gy and seeds for our selected intermediate-risk prostate cancer patients. Within our analysis 78 Gy provides a slightly better biochemical control as the other treatment modalities, although without a significant difference. This might be partly due to the fact that 21% our 78 Gy patients had a Gleason score of 7b, compared to 10% and 16% of our 74 Gy and seeds patients, respectively. Regarding the univariate analysis, we could not confirm T‑stage, Gleason score, and iPSA as significant factors, while ADT was confirmed.

The reported bNED rates after seeds for intermediate-risk in the literature range from around 80% to 90% [4, 6, 7, 11] and above [5, 10] after 5 years and about 55% [4] over 75% [11] to around 90% [7] after 10 years. Regarding the 74 Gy group, our bNED rate is 87% after 5 years and 66% after 10 years. These results are better than the reported bNED rates in intermediate-risk patients after 8 years in a large Australian study [22] with 66%. It is, however, noteworthy that this study also used doses with a total of 70 Gy, which is known to be insufficient in intermediate-risk [4] and even low-risk [23, 24]. The reported bNED rate of the CHHiP trial [25] with around 88% after 5 years in the 74 Gy group is pretty close to our results, although the trial included also low- and high-risk patients and did administer ADT in 97% of the cases, which in our case was an important factor regarding bNED. The MRC RT01 trial [26] showed 5 year bNED rates of 71% and 10 year bNED of 55% with 3D conformal radiotherapy and inclusion of low-, intermediate-, and high-risk patients. The results of the 78 Gy group with 92% bNED rate after 5 years and 85% after 9 years are slightly worse than the outcomes reported by Pasalic et al. [27]. However, caution is advised, as only 2 patients in the 78 Gy group have a follow-up this long. Compared to the free rate of 74% after 5 years reported by Peeters et al. [28], our patients show higher bNED rates, most likely due to the fact that Peeters included a large number of high-risk patients. In addition, failure-free rates were defined using the ASTRO definition.

Regarding late side effects, we observed reduced genitourinary side effects and increased gastrointestinal side effects for EBRT in comparison to seeds. This resembles the results in our low-risk collective [3]. For maximum side effects, we noticed a significant increase for late genitourinary side effects in patients treated with 78 Gy compared to 74 Gy. This might be partly explained due to our 78 Gy patient collective being the oldest one with a median age of 74 years, compared to 73 years in the 74 Gy group and 69 years in the seeds group. Therefore, late GU toxicities for seeds patients might also be underestimated. For late gastrointestinal side effects, we were not able to record a significant difference, possibly due to reduced dorsal PTVs in patients treated with 78 Gy. The alternating but not decaying amount of genitourinary grade 2 toxicity in EBRT might be the result of our patient collective getting older and therefore intrinsically developing more genitourinary problems that might be classified as toxicity.

The main weakness of our study is the retrospective nature and the high proportion of patients with missing follow-up, resulting in median follow-up rates of only 54 months. However, in terms of biochemical control, the follow-up is sufficiently long. Another potential weakness is the choice of treatment modality by the patient, leading to a possible selection bias. On the other hand, our study shows several strengths. As a monocentric study, reporting of side effects is similar of the observed period. Having all patients with seeds treated by only one radiation oncologist allows to assume a high level of quality in treatment, as displayed by Nakamura et al. [29]. Furthermore, our collected data originated from daily clinical practice.

## Conclusion

Regarding bNED rates, no significant difference between seed brachytherapy and external beam radiotherapy (74 or 78 Gy) could be detected in our selected patient collective. Concerning late side effects, less genitourinary, and higher gastrointestinal side effects are observed in EBRT.
